# Supplementary material for: Clinical Significance of Preoperative Hematological Parameters in Patients with D2-Resected, Node-Positive Stomach Cancer
Source: Biomedicines. 2022 Jun 30;10(7):1565. doi: 10.3390/biomedicines10071565 (PMC9312951; doi:10.3390/biomedicines10071565)
Supplement: Supplementary file 1 [file biomedicines-10-01565-s001.zip › biomedicines-1705980-supplementary.pdf]

Supplementary Table S1. Patient characteristics before the propensity score matching.

Variables	All patients (n = 998)	CCRT (n = 579)	Chemotherapy alone (n = 419)	p-value
Age				
≤ 65	785 (78.7%)	515 (88.9%)	270 (64.4%)	< 0.001*
> 65	213 (21.3%)	64 (11.1%)	149 (35.6%)	
Sex				
Male	629 (63.0%)	367 (63.4%)	262 (62.5%)	0.782
Female	369 (37.0%)	212 (36.6%)	157 (37.5%)	
T stage				
T1-2	376 (37.7%)	200 (34.5%)	176 (42.0%)	0.007*
T3	370 (37.1%)	213 (36.8%)	157 (37.5%)	
T4	252 (25.3%)	166 (28.7%)	86 (20.5%)	
N stage				
N1	340 (34.1%)	172 (29.7%)	168 (40.1%)	< 0.001*
N2	315 (31.6%)	166 (28.7%)	149 (35.6%)	
N3	343 (34.4%)	241 (41.6%)	102 (24.3%)	
Stage				
IB-II	452 (45.3%)	230 (39.7%)	222 (53.0%)	< 0.001*
III	546 (54.7%)	349 (60.3%)	197 (47.0%)	
Lauren classification				
Non-intestinal	652 (65.3%)	396 (68.4%)	256 (61.1%)	0.017*
Intestinal	346 (34.7%)	183 (31.6%)	163 (38.9%)	
Surgical extent				
STG	707 (70.8%)	409 (70.6%)	298 (71.1%)	0.868
TG	291 (29.2%)	170 (29.4%)	121 (28.9%)	
Mean ANC (/μL)	3757.2	3749.1	3768.3	0.840
Mean ALC (/μL)	2119.6	2159.2	2064.9	0.021*
Mean AMC (/μL)	456.3	456.6	455.8	0.944

Mean PC (× $10^3/\mu L$)	250.1	253.2	245.9	0.086
Mean NLR	1.952	1.880	2.051	0.044*
Mean LMR	5.130	5.212	5.017	0.129
Mean PLR	129.4	126.6	133.2	0.080

CCRT, concurrent chemoradiotherapy; STG, subtotal gastrectomy; TG, total gastrectomy; ANC, absolute neutrophil count; ALC, absolute lymphocyte count; AMC, absolute monocyte count; PC, platelet count; NLR, neutrophil-to-lymphocyte ratio; LMR, lymphocyte-to-monocyte ration; PLR, platelet-to-lymphocyte ratio.

Asterisks (*) indicate $p < 0.005$.

Supplementary Table S2. Comparison of patient characteristics according to absolute lymphocyte counts.

Variables	All patients (n = 692)	ALC ≤ 2074.0 /μL (n = 347)	ALC > 2074.0 /μL (n = 345)	p-value
Age				
≤ 65	550 (79.5%)	271 (78.1%)	279 (80.9%)	0.367
> 65	142 (20.5%)	76 (21.9%)	66 (19.1%)	
Sex				
Male	417 (60.3%)	187 (53.9%)	230 (66.7%)	0.001*
Female	275 (39.7%)	160 (46.1%)	115 (33.3%)	
T stage				
1-2	288 (41.6%)	142 (40.9%)	146 (42.3%)	0.773
3	262 (37.9%)	130 (37.5%)	132 (38.3%)	
4	142 (20.5%)	75 (21.6%)	67 (19.4%)	
N stage				
1	266 (38.4%)	129 (37.2%)	137 (39.7%)	0.171
2	248 (35.8%)	118 (34.0%)	130 (37.7%)	
3	178 (25.7%)	100 (28.8%)	78 (22.6%)	
Stage				
IB-II	362 (52.3%)	175 (50.4%)	187 (54.2%)	0.321
III	330 (47.7%)	172 (49.6%)	158 (45.8%)	
Lauren classification				
Non-intestinal	458 (66.2%)	236 (68.%)	222 (64.3%)	0.308
Intestinal	234 (33.8%)	111 (32.0%)	123 (35.7%)	
Surgical extent				
STG	479 (69.2%)	235 (67.7%)	244 (70.7%)	0.392
TG	213 (30.8%)	112 (32.3%)	101 (29.3%)	
Adjuvant treatment				
CCRT	346 (50.0%)	163 (47.0%)	183 (53.0%)	0.110
Chemotherapy alone	346 (50.0%)	184 (53.0%)	162 (47.0%)	

ANC (/μL)				
≤ 3448.5	346 (50.0%)	192 (55.3%)	154 (44.6%)	0.005*
> 3448.5	346 (50.0%)	155 (44.7%)	191 (55.4%)	
AMC (/μL)				
≤ 422.5	346 (50.0%)	211 (60.8%)	135 (39.1%)	< 0.001*
> 422.5	346 (50.0%)	136 (39.2%)	210 (60.9%)	
PC (× 10^3/μL)				
≤ 240.5	346 (50.0%)	179 (51.6%)	167 (48.4%)	0.403
> 240.5	346 (50.0%)	168 (48.4%)	178 (51.6%)	
NLR				
≤ 1.657	347 (50.1%)	104 (30.0%)	243 (70.4%)	< 0.001*
> 1.657	345 (49.9%)	243 (70.0%)	102 (29.6%)	
LMR				
≤ 4.956	346 (50.0%)	224 (64.6%)	122 (35.4%)	< 0.001*
> 4.956	346 (50.0%)	123 (35.4%)	223 (64.6%)	
PLR				
≤ 117.6	346 (50.0%)	84 (24.2%)	262 (75.9%)	< 0.001*
> 117.6	346 (50.0%)	263 (75.8%)	83 (24.1%)	

ALC, absolute lymphocyte count; STG, subtotal gastrectomy; TG, total gastrectomy; CCRT, concurrent chemoradiotherapy; ANC, absolute neutrophil count; AMC, absolute monocyte count; PC, platelet count; NLR, neutrophil-to-lymphocyte ratio; LMR, lymphocyte-to-monocyte ration; PLR, platelet-to-lymphocyte ratio

Asterisks (*) indicate $p < 0.005$.
